# Locating and Imaging Fiber Breaks in CFRP Using Guided Wave Tomography and Eddy Current Testing

**DOI:** 10.3390/s22197377

**Published:** 2022-09-28

**Authors:** Dario J. Pasadas, Mohsen Barzegar, Artur L. Ribeiro, Helena G. Ramos

**Affiliations:** Instituto de Telecomunicações, Instituto Superior Técnico, Universidade de Lisboa, 1049-001 Lisbon, Portugal

**Keywords:** guided wave testing (GWT), eddy current testing (ECT), CFRP, fiber breaks

## Abstract

In this paper, guided Lamb wave tomography and eddy current testing (ECT) techniques were combined to locate and evaluate fiber breaks in carbon-fiber-reinforced plastic (CFRP) structures. Guided wave testing (GWT) and computed tomography (CT) imaging were employed to quickly locate fiber breaks in the CFRP plate. From B-scans performed along two different fiber orientations (0 and 90 degrees), parallel-beam projections of different features were extracted from the guided wave signals, using signal-processing techniques (such as wavelet and Hilbert transforms) and statistical functions (such as skewness and kurtosis). The parallel-beam projections of each individual feature were used as input in computed tomography imaging reconstruction to approximately estimate the location of fiber breaks. From the obtained reconstructed images, image-fusion techniques were applied to get complementary information from multiple source images into one single image. After locating the fiber breaks, C-scans were performed in the vicinity of the damage, using an ECT probe with double excitation configuration to evaluate the condition of the fiber break.

## 1. Introduction

Nowadays, carbon-fiber-reinforced plastic (CFRP) materials are widely used in several engineering applications due to their combined advantages of high strength-to-weight ratio and stiffness (rigidity). However, during the service time, those characteristics may be lost if defects are present in the CFRP specimen. Hence, the development of nondestructive testing and evaluation (NDT&E) methods for the detection, location, and characterization of damages, such as fiber breaks delamination, porosity, matrix crack, debonding, or void, in CFRP materials is therefore important.

Various NDT techniques have been developed to evaluate the condition of composite structures, including ultrasonic C-scans [1,2], thermography [2,3] and eddy current testing [4,5]. Each NDT technique has advantages and limitations in the detection of different types of damages. Eddy current testing (ECT), which is based on the principle of electromagnetic induction, has been proven to be an effective NDT technique for the thickness measurement, detection, and characterization of defects in conductive plates [6,7,8]. In the case of a CFRP specimen, the electrical conductivity of the carbon fibers can be used by an ECT System for the detection of damage that causes an electrical conductivity change, as in the case of fiber breaks. The low-conductivity characteristic of the CFRP material requires the use of highly sensitive eddy current probes working at higher testing frequencies, when compared with traditional ECT, to evaluate the condition of the structure [9,10,11]. The ECT probes are generally used to inspect local areas, and ECT Systems are usually performed through a C-scan inspection to evaluate the condition of the material. However, this method can be time-consuming because the inspection of the structure needs to be implemented point by point. So, this method becomes very expensive if it is used to scan large composite structures, such as airplane wings. To reduce the time it takes to perform those C scans, a fast estimation of the damage location is important.

Ultrasonic-guided-waves testing offers a promising alternative for damage detection in composite structures. This method can inspect the condition of the structures without requiring a point-to-point scan. It also has the advantage of not requiring direct access to the region to be inspected, and it uses the propagation of guided waves in solid media with its boundaries as waveguides to evaluate the integrity of the structure. Among guided waves, Lamb waves can cover the total thickness of the materials and can travel long distances inside plates or pipes with relatively low attenuation [12,13]. Along with the greater potential that this method offers, it also comes with an increased level of complexity in the analysis of the received signals due to the dispersive and multimodal nature of the waves. To analyze the Lamb wave signals, it is common to apply signal-processing techniques in time, frequency, and in time–frequency domains to extract useful information from the Lamb-wave signals. Different strategies have been proposed in the literature to detect, locate, and quantify the severity of defects by using guided-wave-testing signals. Machine learning, tomography reconstruction, and triangular and probabilistic approaches are some of those strategies [14,15,16]. Among the subject of defect detection in composite structures, ultrasonic guided waves have been successfully used to detect impact damage [17,18], delamination [17,18], and debondings [19,20]. However, the estimation of the severity of the damages remains a complex task, and it usually requires an increasing number of sensors.

To reduce the complexity in the analysis of the received signal, excitation methods to select desired modes have also been proposed in the literature. One of the methods is based on changing the polarization of piezoelectric transducers, which are usually attached on both sides of the specimen, in order to predominantly excite a desired mode [21,22].

Another method to generate selective modes is using a longitudinal wave transducer fixed in a coupling plexiglass wedge, with a fixed incidence angle to predominantly excite one desired mode [23,24,25]. The angle of incidence should be chosen based on Snell’s law, considering the wave velocity of the coupling medium and the phase velocity of the desired Lamb wave mode. Considering the wave-propagation characteristics, a second transducer, the receiver, should be placed at an appropriate distance, with the same angle as the transmitter.

This paper presents a two-step process to locate and evaluate fiber breaks in CFRP specimens by using guided wave testing and eddy current testing. In the first step, a strategy based on guided wave testing (GWT), computed tomography (CT), using inverse Radon transform and data fusion, was implemented to quickly localize a fiber break in an area of 230 × 230 mm^2^. In this step, an adjustable pair of angle-beam transducers, mounted in a pitch–catch configuration, was used to selectively generate the S0 mode in the CFRP plate. Instead of using a high number of angle projections for the CT imaging reconstruction, the localization process was simplified by considering only two perpendicular projections along two different fiber directions. To evaluate the geometrical characteristics of the damage, a second step involving ECT inspection in the vicinity of the estimated damage location was performed by using our developed ECT probe, with a double-excitation configuration. Section 2 describes the characteristics of the CFRP specimen used to perform the experimental tests. Section 3 describes the first step involving the guided wave testing and the damage localization strategy to localize the fiber break in the inspected region. Section 4 describes the second step, involving the eddy current testing inspection and the characterization of the fiber break by 2D images obtained with the ECT probe. Conclusions and future work are drawn in Section 5.

## 2. Characteristics of the Specimen

**Figure 1:**
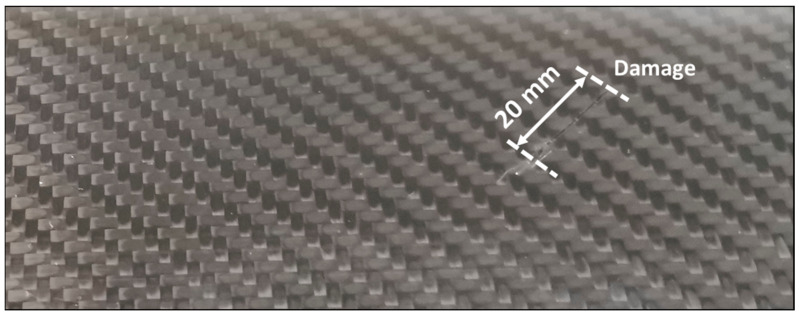
Photograph of a portion of the CFRP specimen containing the artificial damage.

The experimental specimen was a CFRP plate with dimensions of 500 × 470 × 1 mm^3^. It includes fiber layers oriented along 0°/90° and 45°/−45°. This layup enhances the stiffness across the diagonal axis of the CFRP specimen. The mechanical proprieties of the specimen after it was cured are listed in Table 1. One artificial fiber break was created with the length of 20 mm by cutting a portion of fibers in the specimen. Figure 1 depicts a photograph of a portion of the CFRP plate containing the artificial damage.

## 3. Locating Fiber Breaks Using Guided Wave Tomography

### 3.1. Experimental Setup for Guided Wave Testing

**Figure 2:**
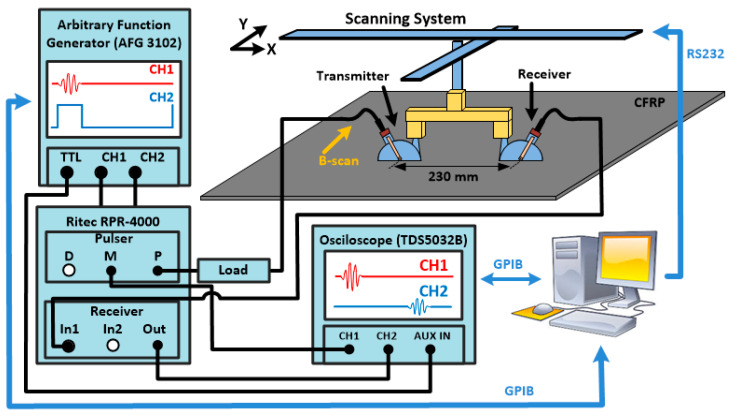
Experimental setup for guided wave testing, depicting the B-scan at 90°.

An initial experimental setup was mounted to detect fiber breaks in a defined area, using guided Lamb-wave testing and tomography-imaging reconstruction. The setup to collect the guided wave signals is depicted in Figure 2. A pair of angle-beam transducers (A413S-SB from Olympus) with variable angle wedge was attached to a two-axis positioning system in a pitch–catch configuration. The central frequency of the transducers is 0.5 MHz. The wedge used to launch the lamb wave into the plate is made of Plexiglas, with a longitudinal wave velocity (C_w_) of 2720 m/s. The two angle-beam transducers (ABT) were fixed, with the distance between them equal to 230 mm. The support used to attach the two ABT to the positioning system allows the manual rotation of the probes in the x–y plane. Water was used as a couplant between the ABT wedge and the test object. For the transmitted signal applied to the first transducer, an arbitrary function generator (AFG3102 from Tektronix) was used to generate a tone burst with five-cycle sinusoid in a Hann window envelope. The output of the AFG was connected to a high-power RPR-4000 pulser-receiver (from RITEC) to excite the signal to the transducer responsible for the transition of the wave into the wedge. Due to the impedance characteristic of the piezo-electric transducers, this transducer was connected to the output of the high-power pulser board through a 150 ohm high-power load (RT 150). The RF pulse-monitor access point (M) was used to acquire the signal transmitted by the first angle-beam transducer through CH_1_ of the oscilloscope.

The second angle-beam transducer was connected to the input of the pulse receiver board to amplify and de-noise the received signal before acquisition. The signal-to-noise-ratio of the receiver signal was increased by adjusting the internal high-pass and low-pass filters of the instrument to 200 kHz and 5 MHz, respectively. After the bandpass filtering process, the received signal was acquired by the oscilloscope through CH2. Examples of the transmitter and receiver signals are depicted in Figure 6.

### 3.2. Selection of the Frequency and the Incident Angle of the Excitation Source

The central frequency of the excitation source was chosen by considering the following six conditions: (1) the central frequency of the transducers; (2) the number of generated Lamb-wave modes to reduce the complexity of the received signals; (3) the selection of a mode with low dispersion in the excitation frequency bandwidth to maintain the waveform independent of the propagation distance; (4) the selection of testing frequencies in which the group velocity of the desired mode is as far as possible from the other modes; (5) the selection of a Lamb-mode wavelength that is lower than the size of the fiber breaks to be detected by the received signals; and (6) the adjustment of the angle-beam transducers of the transmitter and receiver to maximize the transfer of the desired mode to the plate.

**Figure 3:**
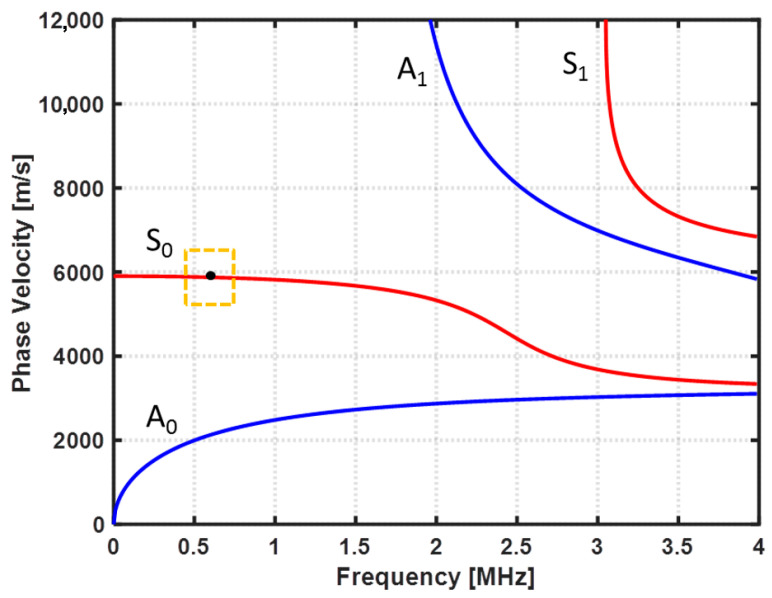
Phase-velocity dispersion curves.

**Figure 4:**
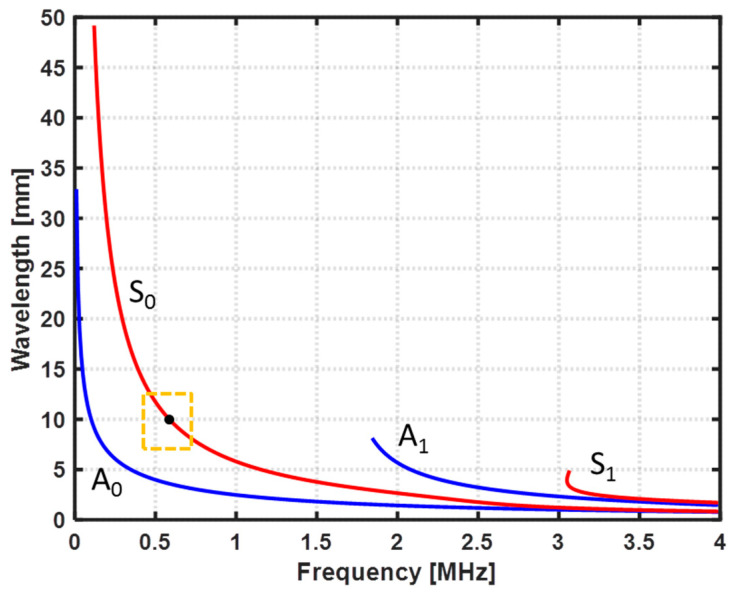
Wavelength for each mode.

To estimate an appropriate testing frequency to inspect the CFRP specimen, the dispersion curves of the CFRP specimen were computed accordantly to the mechanical proprieties of the specimen presented in Table 1, using the Dispersion Calculator software v2.0 created by Armin Huber (Augsburg, Germany) [26]. The obtained plots of the phase velocity (Cp) and the wavelength (λ) are depicted in Figure 3 and Figure 4, respectively. Based on the first five criteria mentioned above and the results obtained with the Dispersion Calculator software, a central testing frequency (f) of 0.62 MHz was estimated to be suitable for the detection of fiber breaks in the CFRP specimen.

**Figure 5:**
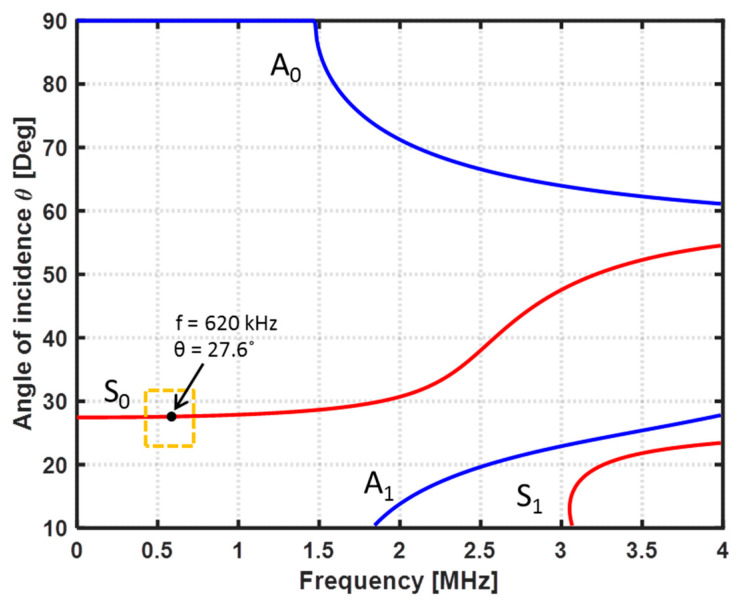
Angle of incidence in the wedge for each mode.

Considering the phase-velocity dispersion curves, and based on Snell’s law, θ = arcsin(Cw/Cp), the incident angles (θ) for each mode were computed and plotted (Figure 5). The incident angle of the transmitter was set to 28 degrees to generate the desired S0 mode at a frequency of 0.62 MHz. The angle of the receiver was also adjusted to 28 degrees.

### 3.3. Experimental Measurements and Feature Extraction

**Figure 6:**
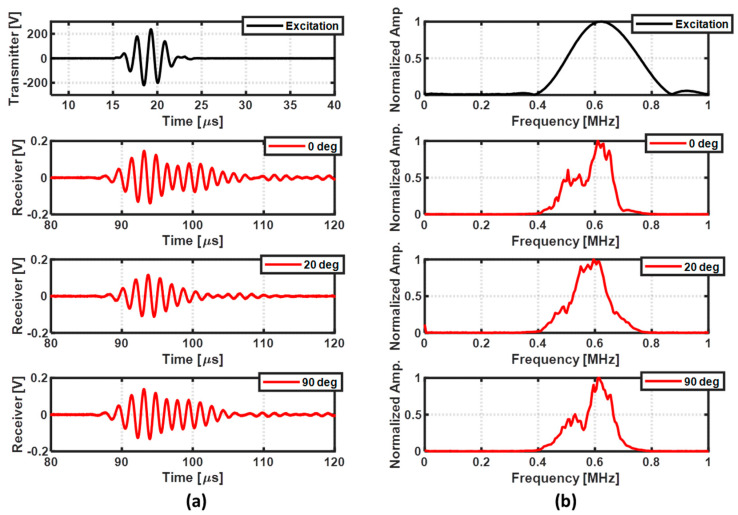
Acquisition of the transmitter and receiver signals when the direction of the pair of transducers was set to 0°, 20°, and 90° in a damage−free region: (**a**) time domain signals and (**b**) normalized amplitude spectrum.

A tone burst consisting of a five-cycle sinusoid with Hann window envelope and maximum amplitude of 200 V was generated as an excitation source. For each position of the transducers, a signal averaging of 20 measurements was performed through the oscilloscope averaging mode to increase the signal-to-noise ratio. For a fixed distance of 230 mm between the pair of transducer pair oriented along three different directions, Figure 6a depicts the results obtained by the transmitter and receiver transducers, and Figure 6b depicts the normalized amplitude spectrum of the signals.

**Figure 7:**
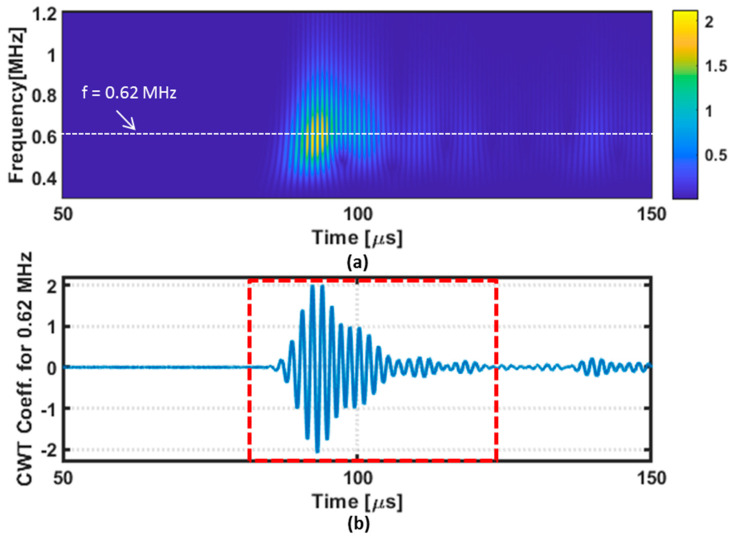
Example of the CWT coefficients: (**a**) CWT coefficients along the time−frequency range and (**b**) CWT coefficients at 0.62 MHz.

It is possible to observe from Figure 6 that the receiver signals vary when the direction of the pair of transducers changes. This could be due to the existence of other paths for the wave propagation other than the shortest path in the composite specimen. However, the receiver signals show a similar envelope when the pair of transducers was fixed along two perpendicular fiber orientations (0° and 90°). Thus, the pair of transducers was fixed along these two directions to collect the GWT data for damage localization. It can also be observed from Figure 6b that the receiver signals contain a large band around the central testing frequency of 0.62 MHz. Hence, the guided wave signals were decomposed into the time–frequency domain by using a continuous wavelet transform (CWT). The wavelet transform was computed with a Morlet mother function because its shape is like the shape of the five-cycle tone burst, with Hann window envelope used in this work as the excitation source. As an example, the result of the decomposition of the receiver signal when the direction of the pair of transducers is at 90° is presented in Figure 7a. From the time–frequency map, a portion of the obtained wavelet coefficients at 0.62 MHz was extracted for feature extraction, as depicted in Figure 7b.

Features were computed to evaluate the peak and shape of the selected CWT coefficients at 0.62 MHz. Table 2 describes the extracted features considered in this study. The symbol $x_{i}$ is the portion of the CWT coefficients used to compute the features for *i* = 1, 2, …, *n*, where n is the total number of points in the analyzed portion. The Hilbert transform (HT) technique was employed to compute the envelopes of the signal to extract the maximum peak of the coefficients. Feature F_1_ contains the information about the peak variation of the wave packet, feature F_2_ contains the information about the asymmetric change of the wave packet, and feature F_3_ contains the information about the change of the spreading of the wave packet when the path between the two transducers is crossing the damaged region.

### 3.4. GWT Scan Procedure

**Figure 8:**
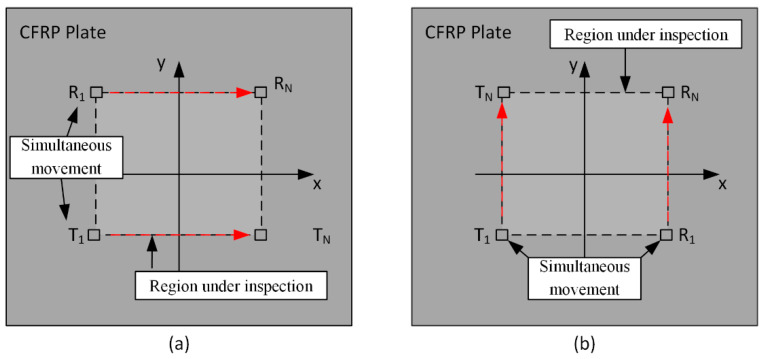
Representation of the two scans used to collect data for the guided-wave-tomography reconstruction: (**a**) B−scan performed along the fiber oriented at 0 degrees and (**b**) B−scan performed along the fiber oriented at 90 degrees.

Figure 8 shows the representation of the scan procedure used to collect data for the guided-wave-tomography reconstruction. Two parallel scans (B-scans) were performed along two perpendicular fiber orientations to localize the presence of a fiber break in a square region of 230 *×* 230 mm^2^. For each B-scan, the two angle-beam transducers were simultaneously moved along the red line, starting from point 1 to point N, and each B-scan was performed by using N = 231 points, with steps of 1 mm. For each movement of the transducers, guided wave signals were transmitted into the plate by the angle-beam transducer (T) and were received by the angle-beam transducer (R), with time intervals of 10 ms.

### 3.5. Method Used to Locate Fiber Breaks

**Figure 9:**
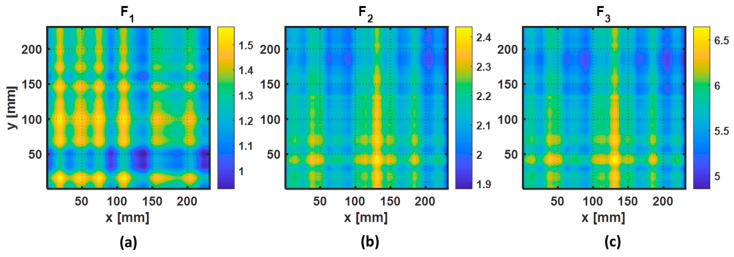
Reconstructed images obtained through the inverse radon transform, using the following features as projection: (**a**) feature F_1_, (**b**) feature F_2_, and (**c**) feature F_3_.

In this work, a computed tomography (CT) imaging reconstruction was used to quickly detect and locate the presence of a fiber break in the CFRP specimen. Based on the guided wave signals collected from two perpendicular B-scans, parallel projections of three features, referred to in Section 3.3, were obtained and used as input for the CT image reconstruction. From each group of parallel projections of one feature, an image was reconstructed by using the inverse Radon transform (IRT). For each individual feature, the reconstructed images obtained by the IRT are depicted in Figure 9a–c. From the reconstructed images obtained by the individual feature, the result shows that the fiber break can be detected in the monitored region. However, the quality of the estimation of the location of the fiber break is not equal when using different features. In Figure 9a, we can see that the attenuation of the peak of the wave packet (F_1_) is visible when the path between the two transducers is crossing the damaged region. In the case of Figure 9b,c, we can see that positive asymmetric and spreading changes (F_2_ and F_3_) of the wave packet were obtained when the path between the two transducers was crossing the damaged region.

To evaluate the performance of each individual reconstructed image with respect to the detectability of the fiber break, quantitative results of each individual GWT feature were obtained. For each individual reconstructed image, knowing the position of the fiber break and the defect-free region, we computed the positive or negative maximum variation (IRT_variation_) that occurred in the fiber-break region and the average value (IRT_avg_) that occurred in a small portion of the defect-free region, and these are presented in Table 3. When the path between the two transducers is crossing the fiber-break region, the results indicate that the change in the spreading of the received signal (F_3_) is higher when compared with the other two features.

**Figure 10:**
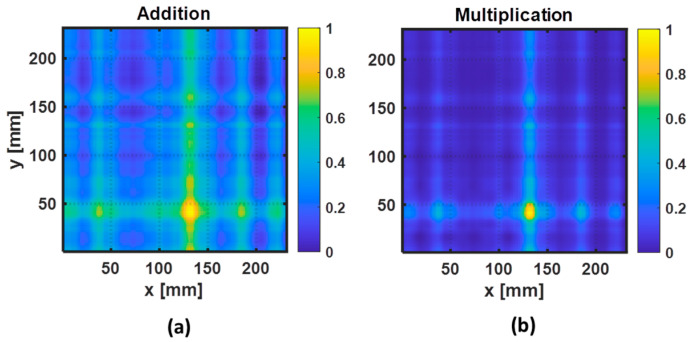
Reconstructed images obtained after the fusion of the three images: (**a**) F_1_+F_2_+F_3_ and (**b**) F_1_·F_2_·F_3_.

Based on the three reconstructed images obtained for each individual feature case, two fusion strategies (addition and multiplication) were also employed to combine the results of F_1_, F_2_, and F_3_. However, before fusion of the three IRT images, each map was normalized. As the feature F_1_ exhibited inverted perturbations with respect to the other two features when the path between the two transducers was crossing the damaged region, the map was inverted before normalization. The resulting images of the data fusion are depicted in Figure 10a,b. From the two fusion strategies, the multiplication of the reconstructed images obtained by three features, using the Hadamard product, shows to be the best image for the localization of the damage position due to the high signal-to-noise ratio obtained in the resulting reconstructed image.

## 4. Evaluating the Fiber Break Using an ECT Probe

### 4.1. ECT Probe

**Figure 11:**
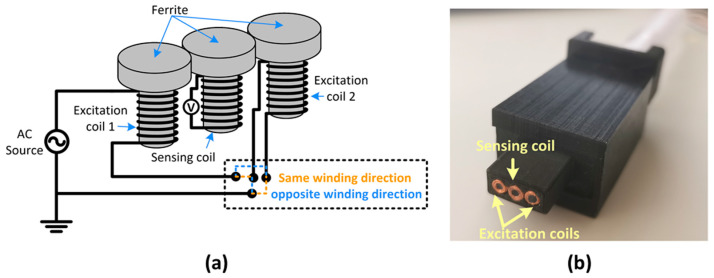
ECT probe used to evaluate the condition of the CFRP specimen: (**a**) schematic and (**b**) photograph.

From the images obtained by the guided wave tomography reconstruction, the fiber break could be quickly detected and localized in the inspected region. However, the geometrical characterization of the fiber breaks could not be evaluated. As a second step of the evaluation process, an eddy current testing probe with double excitation coils and one sensing coil was used to evaluate and estimate the dimensions of the fiber break. Figure 11 shows the schematic and a photograph of the ECT probe that was already used for the detection of damages in another CFRP specimen [11]. This probe allows two different excitation configurations to evaluate the condition of the material by connecting the two excitation coils in series with same or opposite wire directions. The sensing coil was strategically positioned between the two excitation coils to measure the induced voltage that is proportional to the time derivative of the flux linked to the sensing coil. Ferrite cores were used to concentrate the magnetic flux in the center of each coil. When the two excitation coils are connected in a series with the same wire direction, the excitation current orientation causes the eddy currents to flow around the sensing coil and the magnetic field to circulate along the central ferrite core. This excitation configuration is referred to as Configuration 1. On the other hand, when the two excitation coils are connected in series with opposite wire direction, the excitation current’s orientation causes the eddy currents to flow below the sensing-coil region in the CFRP specimen. This excitation configuration is referred to as Configuration 2.

### 4.2. ECT Scan Procedure

**Figure 12:**
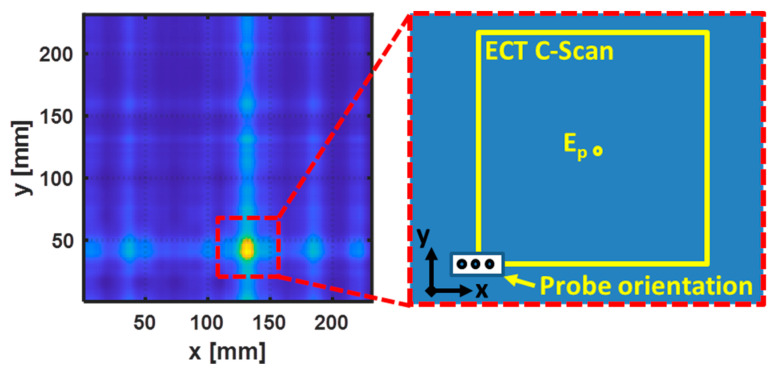
Representation of the C-scan performed in the vicinity of the estimated position of the fiber break.

The ECT probe was attached to the x–y-position system to perform a C-scan around the estimate location of the fiber break, as depicted in Figure 12. The probe was fixed to the scanning system with an air gap of 0.5 mm between the surface of the CFRP specimen and the bottom of the probe. A sinusoidal signal of 10 V of amplitude at the frequency of 0.8 MHz was applied to the excitation coils. The scans were performed with the three coils aligned along the *x*-axis. The positioning system moved the probe with steps of 1 mm in a square region of 50 × 50 mm^2^ around the estimated position of the fiber break (E_p_).

CH_1_ and CH_2_ of the oscilloscope were used to acquire the excitation source and sensing coil voltage. For each position of the probe, a signal averaging of 20 measurements was performed through the oscilloscope averaging mode to increase the signal-to-noise ratio of the two channels. The amplitude of the voltage across the sensing coil and the phase shift between the sensing-coil voltage and the excitation source was extracted by using a three-parameter sine-fitting algorithm [27].

### 4.3. Characterization of the Fiber Break

**Figure 13:**
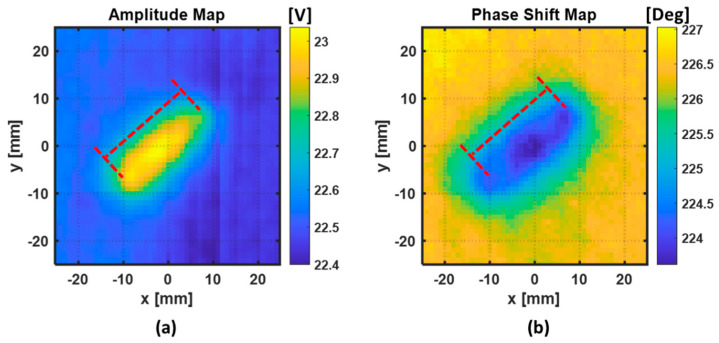
Representation of the C-scan performed in the vicinity of the estimated position of the fiber break, using ECT Configuration 1: (**a**) amplitude perturbations; (**b**) phase shift perturbations.

**Figure 14:**
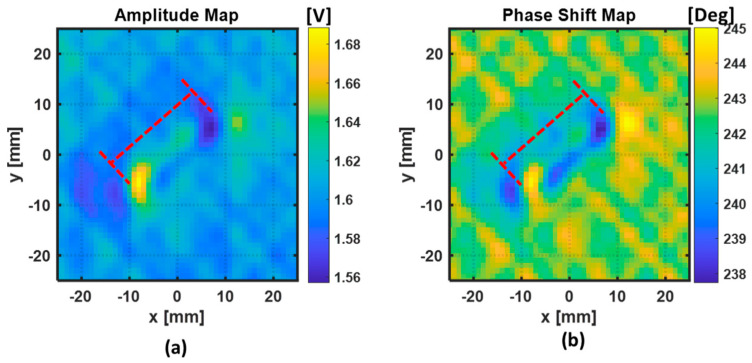
Representation of the C-scan performed in the vicinity of the estimated position of the fiber break, using ECT Configuration 2: (**a**) amplitude perturbations; (**b**) phase shift perturbations.

The 2D maps of amplitude and phase-shift information obtained when the two excitation coils are connected in a series with same wire direction are depicted in Figure 13. The 2D maps of amplitude and phase shift information obtained when the two excitation coils are connected in series with opposite wire direction are depicted in Figure 14. The red dash lines represent the approximate location and dimensions of the fiber break.

From the cases in Figure 13 and Figure 14, it is possible to see that, when the ECT probe is in the proximity of the damaged region, the amplitude and the phase both change. Those variations occur due to the presence of a defect in the material that perturbed the eddy currents from their normal path. In both cases, the orientation of the fiber break was approximately obtained. In the case where the two excitation coils are connected in a series with the same wire direction, as in Figure 13, the amplitude and phase changed when the sensing coil crossed the region of the fiber break. In the case where the two excitation coils are connected in a series with opposite wire direction, as in Figure 14, the amplitude and the phase variations occurred when the sensing coil crosses the edges of the fiber break.

To evaluate the performance of the two ECT probes’ configuration in respect to the detectability of the fiber break, the quantitative results of the two ECT features (amplitude and phase change) are presented in Table 4. The peak-to-peak variations of amplitude and phase in the fiber break region were obtained and referred in this table as A_p2p_ and θ_p2p_, respectively. Knowing the location of the fiber break, we could obtain the average values of amplitude (A_avg_) and phase (θ_avg_) in a small portion of the two maps in the defect-free region.

Knowing that, in Configuration 2, the amplitude and the phase variations occurred when the sensing coil crossed the edges of the fiber break, the length of the fiber break was estimated from the C-scan images (Figure 14). The estimation of the length of the fiber break was performed by estimating the average distance between the positive and negative peaks on either side of the flaw in the amplitude and the phase maps. For the amplitude-map case, the length of the fiber break was estimated to be 23.85 mm, and for the phase-map case, the length of the fiber break was estimated to be 22.47 mm. Comparing the estimated and the real length of the fiber break, it could be observed that the estimated fiber-break length through the phase map was closer to the real length than the estimated fiber-break length from the amplitude map.

## 5. Conclusions and Future Work

In this study, a two-step process, using GWT and ECT, was successfully used to locate and characterize a fiber break that was created in a composite plate. The results suggest that guided wave and eddy current NDT features can be used as complementary information to detect and characterize fiber breaks in composite materials. In the first step, a strategy based on GWT, inverse Radon transform, and data fusion was successfully implemented for the localization of the fiber break in an area of 230 × 230 mm^2^. The advantage of this first step is that it allows a rapid decision for local structural area inspection that would require an intensive evaluation through conventional inspection methods. Despite the characteristics of the CFRP specimen, a good signal-to-noise ratio was obtained in all the receiver signals. A second step involving ECT inspection in the vicinity of the estimated damage location was performed in a small area of 50 × 50 mm^2^ to evaluate the geometrical characteristics of the damage. The results clearly show the detection of the fiber break in the scanned images, using our developed ECT probe with double-excitation configuration. The geometrical characteristic of the fiber break could be observed through the amplitude and phase-shift maps obtained with the two excitations’ configuration of the probe. Using the information of the fiber-break location, we reduced the scanning time of ECT was by 95.27%, when compared with a scanning of the full monitored region.

The proposed strategy of combining two different NDT methods has shown to be more efficient than a full point-by-point NDT inspection of the composite structure. It could be a useful strategy to apply during periodic maintenance of the composite structures. It could also be applied for the in-service inspection of CFRP structures to locate and characterize fiber breaks after an unusual event, such as impact damage or the occurrence of an imbalance load in the structure. However, it is important to mention that both NDT methods have limitations for fiber-break evaluation due to their physical and practical characteristics. As future work, this study can be extended by testing the limitation of the proposed strategy in detecting, locating, and characterizing various fiber-break sizes in the composite structures. In addition, other investigations have shown that guided waves are sensitive to various types of composite damages. Hence, this study can also be extended to detect and characterize different types of composite defects, such as delamination, porosity, matrix crack, debonding, or void. However, in the case of structural damages that do not cause significant electrical conductivity change, the success of detecting and characterizing damages is reduced. Hence, in addition, other local-area methods, such as bulk wave ultrasonic testing, should be considered for the characterization and visualization of the damage in the composite structures.

## Figures and Tables

**Table 1:** Mechanical proprieties of the CFRP specimen.

Parameter	Quantity	Units
Density	1152	Kg/m^3^
Tensile Modulus at 45°	32.3	GPa
Tensile Modulus at 0/90°	37.2	GPa
Poisson ratio	0.33	-

**Table 2:** Features extracted from the obtained CWT coefficients.

Features	Description	Definition
F_1_	Peak	max|HT(x)|
F_2_	Skewness	$\frac{\frac{1}{n}\sum_{i=1}^{n}{\left(x_{i}-\bar{x}\right)}^{3}}{{\left(\frac{1}{n}\sum_{i=1}^{n}{\left(x_{i}-\bar{x}\right)}^{2}\right)}^{3/2}}$
F_3_	Kurtosis	$\frac{\frac{1}{n}\sum_{i=1}^{n}{\left(x_{i}-\bar{x}\right)}^{4}}{{\left(\frac{1}{n}\sum_{i=1}^{n}{\left(x_{i}-\bar{x}\right)}^{2}\right)}^{2}}$

**Table 3:** Quantitative results of each individual GWT feature.

Features	Feature Description	IRT_variation (defect region)_	IRT_avg (defect free region)_
F_1_	Peak	0.42	1.35
F_2_	Skewness	0.28	2.15
F_3_	Kurtosis	0.94	5.70

**Table 4:** Quantitative results of each ECT feature for the two excitation-probe configurations.

Probe Configuration	A_p2p_ (V)	A_avg_ (V)	θ_p2p_ (Deg.)	Θ_avg_ (Deg.)
1	0.64	22.4	7.27	226.41
2	0.13	1.60	3.41	242.71

## Data Availability

The data presented in this study are available upon request from the corresponding author.
